# Impaired angiogenesis in ageing: the central role of the extracellular matrix

**DOI:** 10.1186/s12967-023-04315-z

**Published:** 2023-07-11

**Authors:** Ping Xiao, Yanli Zhang, Yuting Zeng, Dehong Yang, Jiayao Mo, Ziting Zheng, Jilei Wang, Yuxin Zhang, Zhiyan Zhou, Xincen Zhong, Wenjuan Yan

**Affiliations:** 1 Department of Stomatology, Nanfang Hospital, Southern Medical University, Guangzhou, 510515 China; 2 Stomatological Hospital, Southern Medical University, Guangzhou, 510280 China; 3 Department of Orthopedics Spinal Surgery, Nanfang Hospital, Southern Medical University, Guangzhou, 510515 China

**Keywords:** Ageing, Angiogenesis, Extracellular matrix, Therapeutic angiogenesis treatments, Endothelial cell, Pericyte

## Abstract

Each step in angiogenesis is regulated by the extracellular matrix (ECM). Accumulating evidence indicates that ageing-related changes in the ECM driven by cellular senescence lead to a reduction in neovascularisation, reduced microvascular density, and an increased risk of tissue ischaemic injury. These changes can lead to health events that have major negative impacts on quality of life and place a significant financial burden on the healthcare system. Elucidating interactions between the ECM and cells during angiogenesis in the context of ageing is neceary to clarify the mechanisms underlying reduced angiogenesis in older adults. In this review, we summarize ageing-related changes in the composition, structure, and function of the ECM and their relevance for angiogenesis. Then, we explore in detail the mechanisms of interaction between the aged ECM and cells during impaired angiogenesis in the older population for the first time, discussing diseases caused by restricted angiogenesis. We also outline several novel pro-angiogenic therapeutic strategies targeting the ECM that can provide new insights into the choice of appropriate treatments for a variety of age-related diseases. Based on the knowledge gathered from recent reports and journal articles, we provide a better understanding of the mechanisms underlying impaired angiogenesis with age and contribute to the development of effective treatments that will enhance quality of life.

## Introduction

Angiogenesis is the process by which new capillaries form by sprouting from pre-existing ones [1]. This process involves the migration, proliferation, and differentiation of endothelial cells (ECs) and pericytes and results in elongation of the initial tip, followed by anastomosis with other blood vessels to form perfused vascular branches. Accumulating evidence indicates that angiogenesis is impaired in older adults, contributing to cardiovascular and cerebrovascular disease and delayed wound healing, reducing the quality of life and causing a significant burden for healthcare systems [2].

Changes in the extracellular matrix (ECM) may contribute to impaired angiogenesis in older adults. The ECM is a crucial part of the vascular wall and comprises an array of macromolecules, with the two main classes being fibrous proteins (collagen and elastin) and glycoproteins (laminin, fibronectin (FN), and proteoglycans). The ECM plays a crucial regulatory role in all phases of angiogenesis, interacting with cells and acting as a scaffold within which cytokines regulate cell behaviour, promote vascular morphogenesis, and maintain the stability and maturity of the vascular system [3]. Cellular senescence affects transcription, translation, and post-translational modification of ECM components, giving rise to changes in the ECM that can directly or indirectly affect interactions between the ECM and cells and result in impaired angiogenesis.

A summary of the effects of ageing on the ECM and angiogenesis has been lacking to date. In this review, we first present the facts supporting the impairment of angiogenesis with ageing. Then, we summarize in detail ageing-related changes in the composition, structure, and function of the ECM, and explore in detail mechanisms of interaction between ageing-affected ECM and cells during impaired angiogenesis in older adults for the first time. Finally, we address ageing-related diseases involving restricted angiogenesis and propose several novel pro-angiogenic therapeutic strategies targeting ECM, which can provide new insights into the systemic treatment of various age-related illnesses.

## Impaired angiogenesis during ageing

**Fig. 1:**
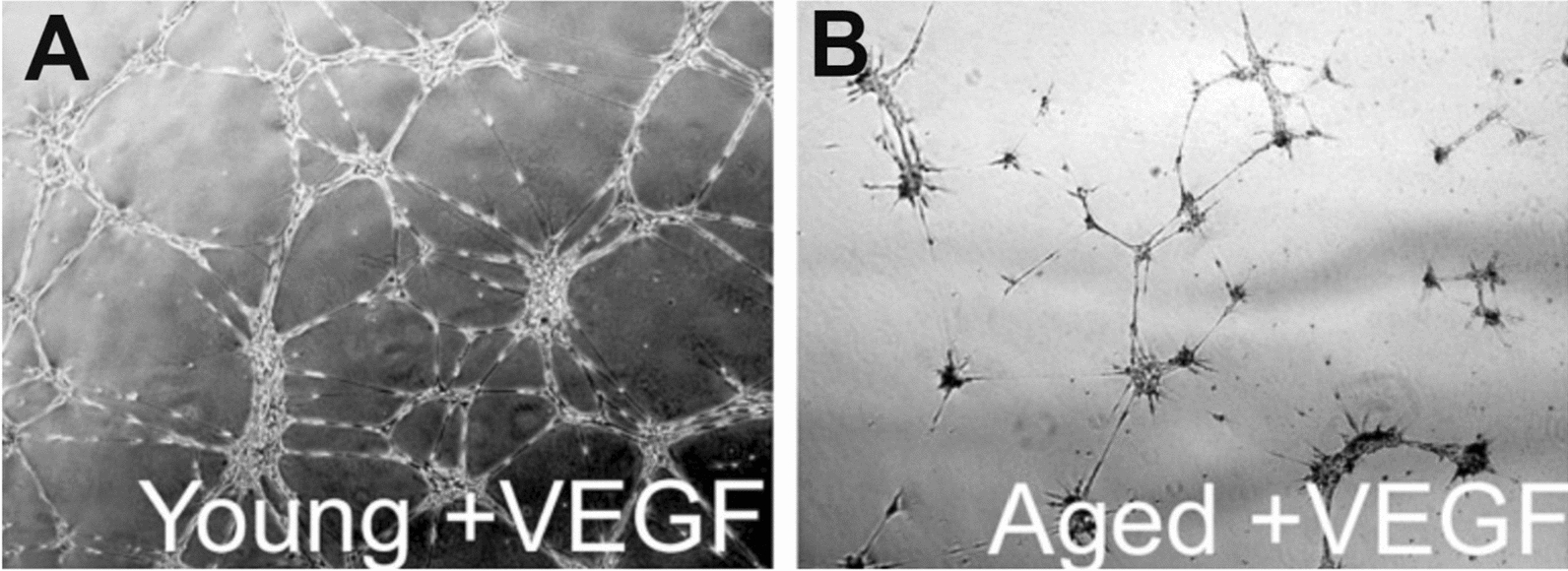
The tube-forming ability of microvascular endothelial cells (ECs) isolated from 3-month-old F344xBN rats (**A**) is greater than that of cells isolated from 24-month-old rats (**B**) [141]

Angiogenesis is a crucial element in wound healing and hemodynamic recovery from ischaemic tissue injury. An increasing number of studies have demonstrated that angiogenic capacity declines drastically with age. Wound healing occurs at a slower rate in older individuals, and a failure of healing may also occur owing to reduced vascular sprouting [4]. In older mice with hindlimb hypoxia/ischaemia, angiogenesis and capillary density at the wound site have been found to be significantly reduced relative to those in younger mice [5, 6]. In older rats, there is a significant decrease in neovascular branching and an increase in the number of free ends in already-formed vascular branches, resulting in a discontinuous vascular network, as seen from studies in vitro (Fig. 1) [7]. In older organisms, the maturation and stability of the neovasculature decrease, the EC lumen width increases, and the vessel structure becomes tortuous [8, 9].

Impaired angiogenesis in older individuals is confirmed by poor blood circulation. In older adults with poor blood flow recovery following acute limb ischaemia, there were considerably higher rates of amputation and mortality [10]. Ageing reduces the endogenous angiogenic response, inhibits the ability to heal injured tissue, and produces ischaemic injury. Ageing and impaired angiogenesis result in higher rates of myocardial ischaemia and infarction, stroke, and peripheral artery disease [11]. An imbalance between myocardial development and angiogenic damage has been shown to be fundamental in contractile dysfunction and heart failure [12]. Additionally, ageing-related angiogenic damage leads to brain microvascular thinning and reduced tissue perfusion, which contribute to the pathogenesis of neurodegenerative disorders, including Alzheimer’s disease (AD). It is clear that ageing has negative effects on angiogenesis, and impaired angiogenesis is a major risk factor for several diseases, including cardiovascular disease. Therefore, understanding the mechanisms of impaired angiogenesis in the context of the development and exacerbation of disease is important for effective management.

## Effects of the ECM on angiogenesis during ageing

The ECM is understood to play an important role in regulating angiogenesis. The ECM binds ECs by interacting with integrins present on the cell surface. This regulates EC activity and initiates vascular sprouting, sending signals to the EC cytoskeleton that promote the formation of vascular cords. ECM remodelling promotes the development of luminal structures and provides pericyte-guiding tunnels to support the construction of the vascular basement membrane, which impacts vascular stability. The ECM also functions as a growth factor reservoir, releasing growth factors in a controlled manner to modulate cellular behaviour during angiogenesis [13]. Hence, the ECM regulates each stage of angiogenesis by influencing the behaviour of cells. The ECM is secreted by cells and surrounds cells within tissues to form the extracellular microecological environment. The structure, composition, and physicochemical characteristics of the ECM change in the context of senescence in cells. Senescent cells can affect the ECM composition, but the ECM has also been shown to regulate senescent cells. Understanding the bidirectional relationship between the ECM and senescent cells is important for comprehending pathological ageing processes. In the following sections, we discuss ageing-related changes in the ECM and the impact of these changes on cells during angiogenesis.

### Changes in the ECM associated with senescent cells

Cellular senescence is a biological process underlying ageing. Factors that contribute to ageing can cause cells to enter a stable and irreversible state of proliferative arrest, characterized by cell cycle arrest, macromolecular damage, metabolic dysregulation, development of a senescence-associated secretory phenotype (SASP), and altered cell morphology [14].

The accumulation of senescent cells directly influences the synthesis, secretion, and remodelling of the ECM. In senescent cells, upregulation of the cell cycle repressor proteins P16 and P21 contributes to arrest in the G1 phase [15], rendering cells incapable of replication and affecting the transcription and translation of ECM genes to cause changes in the composition and structure of the ECM. Fibroblast models showed low expression of mRNA encoding collagen Ia1, Ia3, and IIIa1, accompanied by a significant reduction in collagen synthesis [16]. In senescent parenchymal cells, mitochondrial dysfunction leads to decreased ATP production and generation of reactive oxygen species, leading to oxidation of amino acid side chains, protein cross-linking, and oxidation of the protein backbone, resulting in protein fragmentation. In parallel, proteins can be modified by products of sugar and lipid oxidation [17, 18], leading to dysregulated post-translational modification and altered conformation, which contributes to increased ECM stiffness. Furthermore, metabolic changes in senescent immune cells promote the proliferation of inflammatory macrophages and effector T cells. Matrix metalloproteinases (MMPs), including MMP2 and MMP9, are secreted by inflammatory macrophages, which affect the degradation process of ECM. Senescent cells display the SASP phenotype [14] and secrete pro-inflammatory and catabolic substances, including cytokines, chemokines, growth factors, and MMPs. These substances activate intracellular signalling pathways in an autocrine and paracrine manner, producing an inflammatory microenvironment and initiating angiogenic signalling. Senescent cells affect the structure and content of the ECM through the corresponding secretory phenotype, particularly by releasing MMPs and tissue inhibitors of metalloproteinases (TIMPs), which directly affect ECM degradation (see Table 1 for details). Compared with the young, the MMP/TIMP ratio and net MMP activity of older adults are lower, resulting in a weakening of the degradation ability of ECM, which directly affects the various steps of angiogenesis in older adults [19]. The contribution of MMPs to vascular maturation and stability is a new and innovative concept that will open up a new way to maintain vascular maturation and stability.

**Table 1:** Effect of ageing on angiogenesis-related factors

	Ageing-related change	Impact	Refs.
ECM			
Collagen	Longer and thicker fibrillar structures; formation and cross-linking by AGEs	Decreased integrity of the vascular network and reduced vascular sprouting	[21, 24, 28]
Elastin	Thinner, more fractured, and disordered structure, with a higher degree of calcification	Increased ECM stiffness and interference with angiogenesis	[37–39]
Laminin	Increased *LAMB1* and decreased *LAMB2* expression	Impaired EC formation and migration, disrupting vascular cord formation	[42]
FN	Glycation and changes in globular structure	Increased ECM stiffness, disrupting vascular cord formation	[45, 78]
Perlecan	Decreased levels	Disruption of vascular morphogenesis	[47]
Decorin	Shorter GAG length	Increased ECM stiffness, hindering angiogenesis	[51, 52]
HA	Decreased levels	Increased ECM stiffness, hindering angiogenesis	[47]
MMPs and inhibitors			
MMP-1	Unknown	Unknown	
MMP-2	Increased levels	Endovascular thickening and remodelling	[142]
MMP-3	Unknown	Unknown	
MMP-7	Increased levels	Promotion of fibrosis	[143]
MMP-8	No significant change	–	[143]
MMP-9	Increased levels	Enhanced proliferation and migration of parietal cells; intimal hyperplasia, leading to atherosclerosis; upregulation of endothelial statin, impairing angiogenesis	[144, 145]
MMP-12	Decreased levels	Reducing arterial elastin and altering axial arterial mechanics	[146]
MT1-MMP	Decreased levels	Inhibiting of lumen formation and reducing of pericyte chemotactic proliferation in ECs	[142]
TIMP-1	Increased levels	Inhibiting the activity of MMPs, imbalance of MMPs and TIMPs	[143]
TIMP-2	Increased levels	Inhibiting the activity of MMP-2 and MT1-MMP, impairing ageing-related angiogenesis	[142]
TIMP-3	Increased levels	Inhibiting normal matrix remodelling, limiting neovascularisation	[147]
Angiogenic growth factor			
VEGF	Decreased levels	Imbalance of pro- and anti-angiogenic factors, impairing angiogenesis	[73, 148, 149]
PDGF	Decreased levels		[150]
FGF-2	Decreased levels		[151]
TGF-β	Decreased levels		[152]
Ang-1, -2	No change		[148]
Anti-angiogenic growth factor			
TSP-1	Increased levels	Imbalance of pro- and anti-angiogenic factors, impairing angiogenesis	[73, 153]
TSP-2	Increased levels		[74]
TNF-α	Increased levels		[154]

### Effect of the ECM on angiogenesis

#### Collagen

**Fig. 2:**
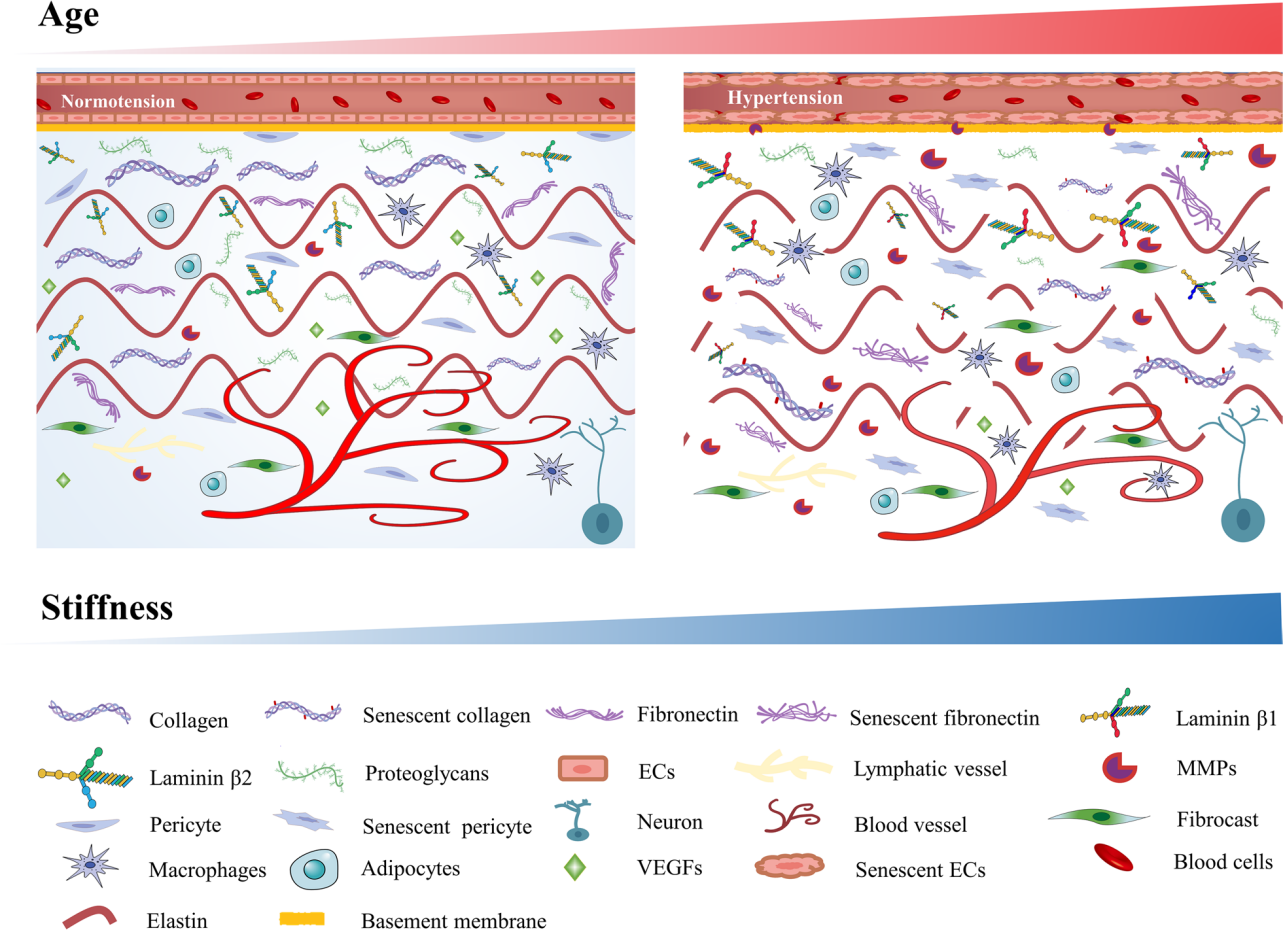
Normal and ageing-affected extracellular matrix (ECM). As compared with normal ECM (left), the composition and structure of ageing-affected ECM (right) are altered, and the matrix stiffness is greater

Collagen comprises approximately 20–40% of the ECM in the vessel wall [20], and it is primarily responsible for vessel mechanical resistance. Ageing changes the structure of the collagen monomer and reduces protofibril assembly (Fig. 2), resulting in the production of a less dense and more heterogeneous structure in older adults [21]. Total collagen density in vessels increases with ageing, despite a decline in collagen synthesis, as collagen has a long half-life [22]. The degradability of collagen declines with time as a result of enhanced resistance to proteolysis due to cross-linking and modification by advanced glycation end-products (AGEs). AGEs reduce cysteine protease-mediated collagen degradation by approximately 20% [23], and these changes limit the rate of neointima formation. Total vessel length was found to increase by approximately 11-, 6-, and fourfold, respectively, when microvascular fragments isolated from rat epididymal fat pads were incubated for 6 days with 2.0, 3.0, and 4.0 mg/mL collagen, at the same time that the integrity of the vascular network decreased with increasing collagen density [24].

Collagen assembly into protofibrils is followed by cross-linking catalysed by lysyl oxidase (LOX) [25, 26]. Increased covalent cross-linking of molecules within collagen protofibrils over time provides a high degree of mechanical and chemical stability in the ECM [27], resulting in a decreased rate of new vessel branch formation and reduced vascular sprouting. Collagen remains in the body for an extended period and is hence prone to ageing-related alterations, such as oxidation, carbonylation, carbamylation, and glycation. Collagen undergoes non-enzymatic modification to form AGEs (Fig. 2), which give rise to cross-linking during ageing [28], forming a network of longer and thicker fibrils [21]. These changes alter the ECM physical and mechanical properties and hence interactions with cells. During the initial phase of elongation, AGE cross-linking increases the tensile Young’s modulus of collagen molecules by 3.0–8.5% [29]; however, AGE accumulation also increases protofibril stiffness [17]. These structural changes alter ECM function and indirectly regulate angiogenesis by modifying interactions with cellular receptors, such as the receptor for advanced glycation end products (RAGE) [30]. This in turn affects the signalling cascade initiated by RAGE, leading to changes in cellular behaviour, phenotype, and the release of inflammatory mediators.

#### Elastin

Elastin is the most abundant ECM protein in the arterial wall, accounting for 50% by weight. It is composed of 90% elastic fibres and 10% microfibrils and provides elasticity and ductility [31]. Across the lifetime, elastin fibres undergo approximately three billion stretch and retraction cycles. Elastin fibres can rupture and fragment under the influence of stress fatigue, post-translational modifications, and calcification [32]. Stress fatigue caused by repeated stretching and retraction can cause elastin to rupture mechanically [33], while post-translational modifications such as glycation and carbamylation can lead to a decrease in elasticity and an increase in stiffness [34]. At a higher degree of elastin calcification, there is a higher risk of rupture, and ageing-dependent changes in the conformation of elastin may give rise to an increased Ca^2+^ content [35, 36]. The binding of Ca^2+^ to elastin reduces elasticity, thereby increasing ECM stiffness and interfering with angiogenesis [37].

The incidence of elastin fracture and degradation remodelling increases with ageing and results in significant morphological and structural changes. Aortic tissue from younger donors has thick parallel elastic fibres, while tissue from older donors shows thinner and fractured elastic fibres (Fig. 2), with the fibrous network topology changing from circumferential to disordered [38]. The elastin-to-collagen ratio decreases with age, which shifts the mechanical load to collagen, which is 100–1000 times stiffer than elastin [33], accounting for increased arterial stiffness [39].

#### Laminin

Laminin is a heterotrimeric glycoprotein composed of α-, β-, and γ-chains [40] and is a modulator of cell migration, differentiation, and adhesion [41]. Laminin β-chain (LAMB) expression is affected by ageing, with *LAMB1* expression increasing and *LAMB2* expression decreasing (Fig. 2). This change in the ratio of laminins β_1_ and β_2_ affects EC function and phenotype. Compared to laminin β_1_, laminin β_2_ activates integrin β_1_ more efficiently to mediate EC adhesion. With ageing-associated changes, integrin β_1_ activation is thus reduced, and short-term adhesion of ECs is reduced, impairing EC migration and blood vessel formation. Compared to ECs cultured on laminin 421 (containing *LAMB2*) substrates, those cultured on laminin 411 (containing *LAMB1*) substrates showed increased expression of mesenchymal markers, including calmodulin, SM22, and vimentin. At the same time, endothelial markers, including VE-cadherin, showed lower expression and fewer tight junctions between ECs [42], a phenotypic change that can disrupt vascular cord formation.

#### FN

FN is a major component of the ECM and is a dimer composed of homologous repeating structural motifs comprising type-I, -II, and -III modules. The soluble dimers fold to form a closed globular structure with binding sites for other ECM proteins and cells [43]. The FN globular structure changes with ageing, and this affects angiogenesis signalling pathways and results in tissue failure. ECM stiffness increases with age; however, the secondary structure of individual FN proteins, when deposited on a rigid matrix, shows more stretching and unfolding when compared with the structure on a soft matrix (Fig. 2). Cells utilize the ageing matrix as a scaffold, and under tension, the FN secondary structure gradually stretches and unfolds, thereby allowing continuous remodelling of the matrix [44]. FN conformational change alters the direction of arrangement of fibres from the original parallel arrangement to an anisotropic form [45], which increases the stiffness of the matrix and affects interaction between cells and the ECM, ultimately affecting vascular cord formation.

In addition, with ageing, FN becomes glycated. Glycated FN has an altered conformation and binds to RAGE, which blocks FN binding to integrins, greatly reducing vascular endothelial growth factor (VEGF)-stimulated signalling (discussed in more detail below), and diminishing the pro-angiogenic effects of FN.

#### Proteoglycans (PGs) and glycosaminoglycans (GAGs)

PGs are complex macromolecules made up of a core protein and one or more covalently linked glycosaminoglycan (GAG) chains. GAGs can be classified based on the core disaccharide units and include heparan sulphate, dermatan sulphate, keratan sulphate, and chondroitin sulphate. Due to their complex composition and capacity to interact with different receptors, heparan sulphate proteoglycans (HSPGs) and chondroitin sulphate proteoglycans have diverse effects on cancer and angiogenesis [46].

Perlecan is a component of HSPG. During ageing, perlecan levels decrease [47], which influences EC activity by altering the activation of pro-angiogenic growth factors and thus influencing vascular morphogenesis. Furthermore, during ageing, the level of GAG sulfation is reduced, which restricts pericyte recruitment and impairs vascular maturation [47, 48].

Decorin is the prototype molecule of the small leucine-rich proteoglycan (SLRP) family [46] and plays an important role in angiogenesis. SLRPs bind to type-I and -VI collagen and FN, control the formation of collagen fibrils, and affect FN stability and biomechanical properties, thus influencing the majority of processes in angiogenesis [49]. The main difference between normal and ageing-affected core proteoglycans in skin tissues is a shorter GAG length in older adults. This is caused by partial GAG degradation in core proteoglycans associated with extracellular glycosidase activity, which reduces the distance between collagen fibrils, reduces grid porosity in the matrix, and limits the flexibility of cells in the ECM [50]. The levels of the core proteoglycan GAG and total amounts of sulphated GAG are much lower in the skin of older individuals than in the skin of younger individuals [51]. These changes alter the assembly of collagen protofibrils, increase ECM stiffness, and hinder angiogenesis [52].

Hyaluronic acid (HA) is the only GAG that is not covalently linked to proteins and acts in angiogenesis primarily by influencing the behaviour of ECs [53, 54]. HA synthesis declines with age [47], and this may influence angiogenesis by increasing ECM stiffness and altering EC binding and proliferation (Fig. 2). Varying HA molecular weights differentially affect angiogenesis; high-molecular-weight HA has an anti-vascular-growth effect, while low-molecular-weight HA has a pro-vascular-growth effect [55]. The effects of ageing on HA metabolism in high- or low-molecular-weight forms and total HA tissue content remain poorly understood. Although the skin of middle-aged rats was shown to contain higher-molecular-weight HA [56], the effect of ageing on the ratios of high- to low-molecular-weight HA has not been determined. It is possible that different organs regulate HA expression differently, and this should be the focus of future research.

Ageing-affected ECM proteins are expected to show higher levels of cross-linking and post-translational modification, resulting in a stiffer matrix and affecting signal transduction in angiogenesis. The conformation of ECM proteins may also change; for example, the globular conformation of FN becomes more expanded, exposing the binding site and affecting vascular sprouting and cord formation. ECM remodelling also becomes unbalanced during ageing, affecting the formation of the vascular lumen and vascular maturation. Epithelial-mesenchymal transformation (EMT) is a trans-differentiation process in which ECs lose unique characteristics during ageing and differentiate to mesenchymal phenotypes with a capability for migration and invasion [57]. EMT is the main driving factor for mechanical and structural changes in the ECM during ageing. The EMT-inducing transcription factor ZEB1 can increase the levels of members of the LOX family of enzymes, especially LOX2, leading to increased cross-linking of collagen fibres in the ECM, which increases matrix stiffness [58, 59]. During angiogenesis, ECs convert to secretory mesenchymal cells with a substantially increased production of ECM [60], activating the hexosamine biosynthesis pathway (HBP) via X-box-binding protein 1 (XBP1), an upstream transcription regulator of EMT and HBP. This then upregulates the N-glycation of ECM proteins [61, 62], protects ECM proteins against hydrolysis, and increases ECM deposition. Upregulation of N-glycosylation promotes the secretion of ECM proteins [61], and leads to further increases in the deposition, cross-linking, and modification of the ECM, resulting in a highly fibrotic microenvironment with increased rigidity. In addition, EMT can also stimulate the production of MMP, tissue remodelling [63], and release of soluble factors, affecting all processes of angiogenesis. EMT affects the mechanical properties of the ECM and is affected by these properties in a feedback loop. For example, increased matrix stiffness can activate transcriptional factors for EMT or promote the nuclear localisation of transcription factors, thereby promoting EMT [64]. The feedback between EMT and ECM is tightly regulated in healthy tissues but is often dysregulated in disease or ageing, and this may contribute to impaired angiogenesis in older adults.

## Mechanisms by which the ECM affects angiogenesis

Interaction between ageing-affected ECM and ECs regulates vascular morphology, and interaction with pericytes can regulate the maturation and stability of blood vessels. In addition, the ECM interacts with immune cells and macrophages in tissues to participate in the regulation of the immune microenvironment in angiogenesis.

### Dynamic crosstalk between ageing-affected ECM and ECs

In older adults, inflammatory factors stimulate the release of pro-angiogenic signals from resting vessels, causing pericytes to be released from the basement membrane by proteolysis catalysed by MMPs, relaxing EC connections, and promoting neovascularisation. ECs are stimulated by ECM signals to proliferate and migrate to develop vascular sprouts and promote neovascularisation. In the earliest stage of vascular morphogenesis, the morphology of ECs changes, and ECs arrange to form cord-like structures with aggregated polygonal networks [65]. Subsequently, under the influence of matrix elements, including collagen I, vacuoles are formed and arranged inside ECs, and then through integrin- and Cdc42/Rac1-dependent endocytosis, the vacuoles in these cells fuse into cellular cavities for initial formation of new vascular cavities [66].

#### Impact on vascular sprouting

Tip cells rely on myosin-II contractility to expand filamentous pseudopod branches and remodel the microtubule cytoskeleton, a process that is dependent on the physical properties of the ECM [67, 68]. In soft, deformable matrices, local downregulation of myosin-II contraction drives platelet pseudopod formation and initiates EC branching [68]. As described, ECM stiffness increases significantly in ageing-affected vessels, which enhances myosin-II activity, increases the growth persistence of microtubules (MT) and decreases the rate of MT assembly. This, in turn, reduces the rate of EC branch formation [67, 69] and reduces the total vessel length formed [70]. The number of free ends on each vascular unit also increases, resulting in the formation of a discontinuous vascular network [24]. Once tip cells are selected, the branching tip EC binds to a more distant ECM via an exploratory lamellar foot. The physical properties of the ECM pull the newly created vascular bud in a new direction, with a stiffer matrix providing a more stable and continuous pull. While the number of newly formed branches is modest in ageing-affected vessels, the directional persistence of formed branches is considerably higher, and EC migration polarity is higher [68]. Beyond the mechanical direction, EC migration is influenced by chemotactic gradients of angiogenic cytokines and other ECM components [71]. Levels of angiogenic cytokines, including VEGF, fibroblast growth factor 2 (FGF-2), platelet-derived growth factor (PDGF), and transforming growth factor β (TGF-β), are downregulated in the ageing body, whereas levels of anti-angiogenic factors, including thrombospondin (TSP) and tumour necrosis factor (TNF)-α, are upregulated. An imbalance between pro- and anti-angiogenic factors thus arises, along with a decrease in cytokine-driven chemotaxis and EC migration [72–74]. Furthermore, independent of cytokines, a gradient of immobilized ECM components can directly promote EC migration [75]. A reduction in the gradient of immobilized ECM components during ageing can thus inhibit EC chemotaxis. Therefore, even though the directional persistence of EC migration is high during angiogenesis in ageing-affected organisms, EC migration is diminished under the combined influence of the mechanisms mentioned above, resulting in reduced vascular sprouting. At the same time, the increased tension allows stem cells (proliferating endothelial cells) to implant into the developing bud and proliferate indefinitely, thereby extending the vascular bud [76, 77].

FN undergoes a non-enzymatic glycation reaction during ageing, and glycated FN readily binds to RAGE, competing with the receptor VEGFR2 for c-Src binding and thereby directly inhibiting the formation of the VEGFR2-c-Src complex and affecting the activation of downstream angiogenic signals, such as those of Rac and MEK, required for EC proliferation. As a result, ageing-affected FN directly inhibits EC proliferation [78].

The ECM plays a role in EC survival and death, and when EC binding to the ECM is lost, focal adhesions and paxillin are degraded, and FAK is inactivated. As a result, RhoA is activated and induces anoikis via a kinase cascade from ROCK to MKK4/MKK7 to JNK. Additionally, inactive FAK prevents activation of the PI3K-AKT signalling pathway, affecting EC proliferation, migration, and survival [79]. EC proliferation, migration, and survival are thus primarily facilitated by EC binding to the ECM during vascular sprouting. Normally, endothelial cells bind to FN in the ECM via α_4_β_1_/α_5_β_1_ integrins, however, during ageing, changes in FN conformation facilitate binding to RAGE, which inhibits FN-integrin binding and disrupts EC-ECM adhesion. Moreover, senescent ECs express approximately 50% lower levels of integrins than younger ECs, and it has been reported that β-subunit maturation in some integrins is defective, resulting in diminished endothelial cell binding to FN [54, 80]. In addition, proteoglycans and type-I collagen play a vital role in adhesion. However, type-I collagen is glycated in the ageing organism, and glycated collagen disrupts EC-ECM adhesion in two ways [81]. First, the EC surface is enriched in PGs, which bind electrostatically to the collagen matrix, but the net positive charge of glycated collagen is reduced by neutralisation of the basic charge of lysine residues, which reduces the electrostatic interaction with anionic PGs [81–83]. Second, EC α_1_β1/α2β1 integrins, which are major receptors for type-I collagen, normally bind to the six-residue sequence GFOGER of collagen, which is about 10 nm away from the main protofibril glycation site [84, 85]. Thus, collagen glycation disrupts integrin-collagen junctions and reduces EC binding to the ECM, resulting in decreased EC proliferation and migration. The motility of ECs cultivated on ageing-affected collagen is reduced by 65%, and this could be a major cause of diabetic retinopathy [83]. Viscoelastic GAGs, including HA, directly mediate EC binding to the ECM; therefore, an ageing-related reduction in HA levels impairs EC-ECM binding [54].

**Fig. 3:**
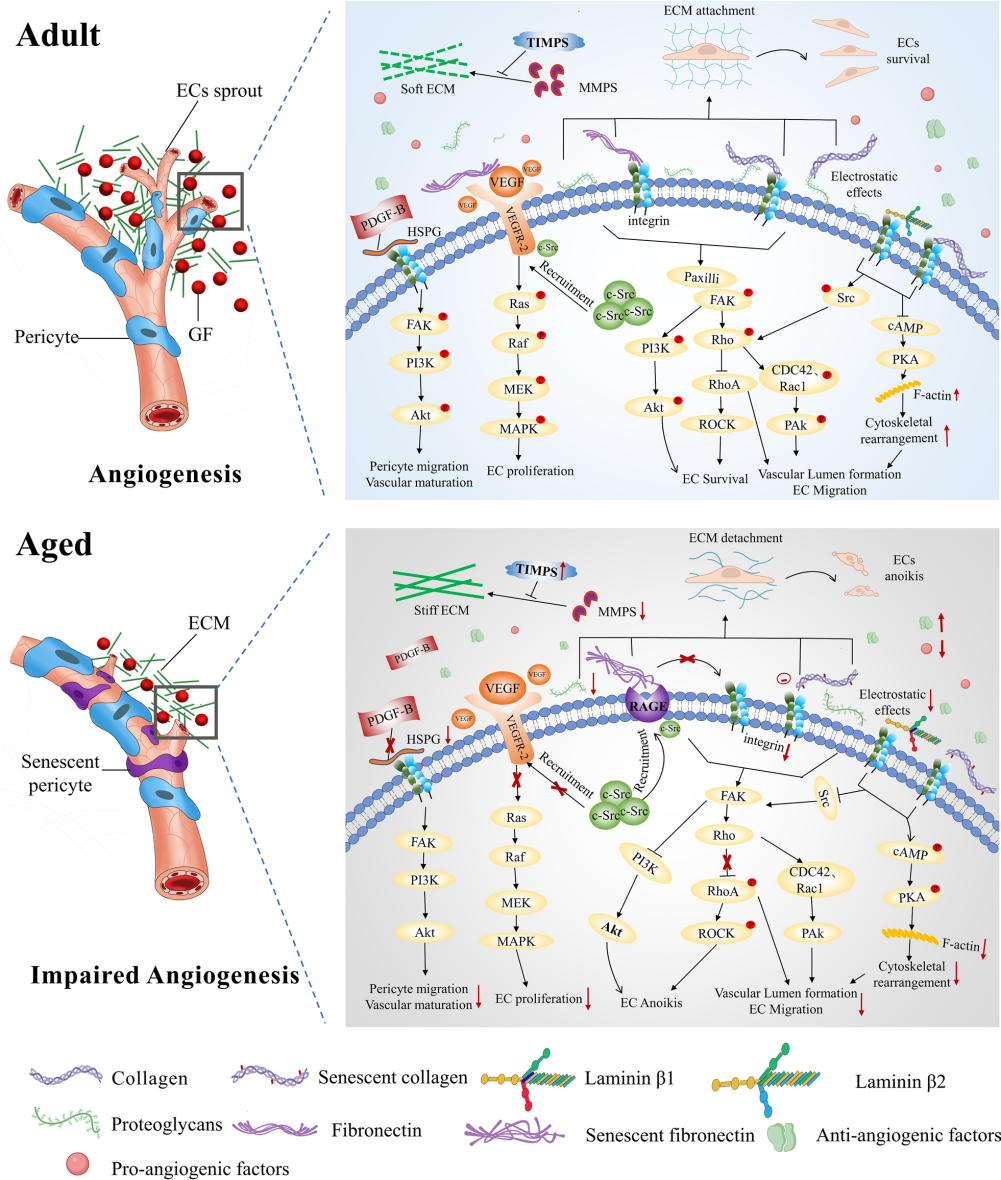
Mechanisms of interaction of ageing-affected ECM with ECs and pericytes during angiogenesis. In younger adult ECM (top), the softer matrix interacts with ECs to promote survival, proliferation, migration, and differentiation, and there is neovascular growth from blood vessels, forming a vascular lumen that interacts with pericytes, leading to pericyte recruitment and promoting neovascular maturation. In ageing-affected ECM (bottom), the rigid matrix interacts with ECs to promote apoptosis and reduce the proliferation, migration, and differentiation capacity of ECs, leading to a decrease in neovascular growth. In addition, the interaction with pericytes leads to a decrease in the capacity for pericyte recruitment and a reduction in the ability of the neovasculature to mature and stabilize

In summary, ageing influences the formation of EC branches as well as EC proliferation, migration, adhesion, and other cellular behaviours by changing the physical properties and composition of the ECM. Ageing also reduces the number of vascular sprouting events, ultimately resulting in impaired angiogenesis (Fig. 3).

#### Impact on the development of vascular cords

The ECM influences vascular cord formation primarily through two mechanisms, the most important of which involves the interaction of the ECM with cell surface integrins via collagen I. During the vascular sprouting phase, ECs degrade the basement membrane and are exposed to the interstitial matrix, binding to type-I collagen via cell surface α_1_β_1_/α_2_β_1_ integrins. cAMP levels drop and hence cAMP-dependent protein kinase A (PKA) activity is inhibited and actin polymerisation induced, leading to the formation of stress fibres that drive EC contraction [86]. Src kinase and GTPase Rh0 are also activated, and this contributes to the formation of actin stress fibres and disrupts endothelial VE-calmodulin linkage complexes among ECs. This, in turn, disrupts intercellular junctions and promotes multicellular reorganisation, whereby ECs rearrange into a cord-like structure [87, 88]. In contrast to the effects of collagen I, the binding of basement membrane laminin-1 (laminin-111) suppresses Src and Rho activity and stimulates persistent activation of PKA and the GTPase Rac to maintain EC integrity, which is important for vascular maturation [89, 90]. In ageing-affected vessels, glycated collagen I affects vascular morphology via disruption of integrin-collagen binding [84, 85], which inhibits the activation of signalling pathways that drive cytoskeletal reorganisation (Fig. 3). Simultaneously, expression of the laminin β chain is altered during vascular ageing, with an increase in *LAMB1* expression and a decrease in *LAMB2* expression, promoting conversion of ECs to a mesenchymal phenotype [42], inhibiting Src/Rho and promoting PKA/Rac activation, resulting in decreased EC contraction, reduced formation of actin stress fibres (Fig. 3), and formation of stable connections between ECs. These changes in ECs are not conducive to multi-cell assembly or rearrangement into vascular cords, but rather facilitate a high degree of vascular maturation and stability [41].

The ECM functions as a malleable 3D scaffold, allowing ECs to orchestrate the construction of multi-cellular structures, an atypical mode of EC intercontact. A guiding path is generated in EC clusters by means of mechanical contractile force with the ECM, and this force is transferred from a single EC to more distant ECs, facilitating the development of vascular cords [91, 92]. However, in ageing vessels, a significant increase in matrix stiffness alters the morphology of endothelial precursor cells from a spindle shape to an isotropic diffuse state [18]. Increased matrix stiffness also activates Rho kinase, leading to myosin phosphorylation and enhanced EC actin-mediated contractility. This contractility is distinct from the collective contraction of endothelial cells in a soft matrix; in a stiff matrix, ECs contract individually [93]. Furthermore, contraction traction is unevenly distributed within the endothelial monolayer, which causes damage to the EC monolayer, resulting in a loss of EC integrity, wider gaps, and poor connections between ECs [18, 93, 94]. This directly affects the transmission of mechanical force within the ECM between EC clusters, weakening the domino effect of force transfer. The endothelial barrier is disrupted by this monolayer driving force mechanism, which makes it impossible to form vascular cord-guided routes between EC clusters. Increased matrix stiffness enhances force transduction between cellular junctional complexes, such as that of VE-calmodulin. The number and size of cell–cell adhesion patches also increases with increased matrix stiffness [93, 94], which stabilizes intercellular connections. Even though the cell–cell connections are not tight, they are relatively stable and difficult to disrupt. Reassembly and the formation of multiple-cell arrangements are thus inhibited, ultimately resulting in a significant reduction in the number of vascular cords created (Fig. 1).

The Cdc42 signalling pathway and membrane type 1 matrix metalloproteinase (MT1-MMP)-dependent protein hydrolysis contribute to the development of luminal structures in ECs; thus, ageing-related alterations in MMPs influence ECM remodelling and directly affect the formation of vascular luminal structures (Table 1).

### Dynamic crosstalk between ageing-affected ECM and pericytes

The EC lumens are initially made up entirely of ECs and are unstable and require mural cell support and ECM redeposition at the periphery before maturing into stable and functioning conduits. The original 3D collagen matrix at the periphery of the EC lumen is degraded by MT1-MMP-mediated protein hydrolysis to generate vascular guidance tunnels and serve as a surface for EC migration, permitting EC movement and MMP-independent lumen remodelling. Simultaneously, factors including PDGF-β recruit pericytes from the surrounding ECM and allow migration along the EC surface outside the artery lumen. The interaction between ECs and pericytes stimulates the deposition of vascular basement membrane matrix proteins, promoting vascular maturation and stabilisation.

#### Impact on vascular maturation

Pericytes are required for the formation of capillary networks. TGF-β1, PDGF-β, angiopoietin (Ang) 1, and sphingosine 1-phosphate are four main molecules that regulate pericyte recruitment. These effectors bind to receptors to promote pericyte proliferation, differentiation, and migration towards tunnels, facilitating vascular maturation and providing a physical template for subsequent cell–cell interactions during vascular stabilisation. The ECM and MMPs play critical roles in these processes. The C-terminus of PDGF-β secreted by ECs comprises a positively charged amino acid sequence that binds to negatively charged sulphate groups on HSPG in the ECM and prolongs retention of PDGF-β outside the cell. This improves pericyte-receptor binding efficiency, promotes pericyte recruitment to the vascular guidance tunnel, and maintains vascular stability to later provide a physical template [95]. During ageing, the content and sulfation of HSPG decrease progressively [48], and this reduces PDGF-β binding and allows PDGF-β to diffuse freely outside the cell, ultimately diminishing the efficacy of contact with the receptor (Fig. 3). In addition, a progressive decrease in levels of PDGF-β receptor in the vascular system of ageing tissues further weakens PDGF-β interaction, which inhibits pericyte recruitment and disrupts basement membrane assembly. The width of EC tubules increases or becomes tortuous, and, as discussed previously, this is a vascular feature of older adults [96].

#### Impact on vascular stability

The deposition of vascular basement membrane proteins, which is required for vascular stability, represents the final key stage in angiogenesis. The expression of integrins α_1_β_1_, α_3_β_1_, α_6_β_1_, and α_5_β_1_ is upregulated and the vascular basement membrane is remodelled via integrin recognition of a variety of matrix elements, including type-IV collagen, laminin, and FN, as well as by membrane protein deposition between cells [97, 98]. In addition, EC-pericyte interactions upregulate the levels of TIMP-2 and TIMP-3. These MMP inhibitors promote vascular basement membrane remodelling by inhibiting MT1-MMP activity and reducing MMP-dependent degradation of ECM components. TIMP-2 and -3 also inhibit VEGF-dependent signalling responses to maintain vascular stability and reduce vascular neogenesis [99].

With ageing, EC expression of integrins decreases by approximately 50%, and defects in β-subunit maturation in integrins have been reported [54]. These defects change basement membrane proteins that are deposited outside the lumen. Changes in collagen levels, increased FN levels, decreased elastic fibre content, and decreased elastin-to-collagen ratios have been observed in older individuals [22, 39, 100]. In addition, laminin-β1 expression increases with age [42]. Changes in ECM protein composition and content affect the mechanical characteristics of the ECM, meaning that the ECM becomes stiff, and since the ECM modulates cellular function, ageing indirectly influences cellular behaviour. In addition, in the microenvironment of ageing vessels, TIMP2 and TIMP3 are significantly upregulated, which leads to a decrease in the activity of MT1-MMP and dysregulation of MMP-mediated proteolysis [101], resulting in a decrease in ECM degradation and an increase in the levels of ECM components deposited on the vascular basement membrane. This is a primary cause of intimal thickening, atherosclerosis, and other diseases, and it shows that ageing indirectly influences or is a risk factor in various pathologies.

### Dynamic crosstalk between ageing-affected ECM and other cells

Fibroblasts are a fundamental cell type that controls ECM remodelling and coordinates angiogenesis. The ECM is also essential in controlling fibroblast behaviour. Increased ECM rigidity, observed with ageing, gives rise to fibroblast activation. Additionally, the interaction of ECM AGEs with RAGE, combined with the upregulation of integrin-mediated mechanotransduction signalling during ageing, results in fibroblast activation and the acquisition of a cancer-associated-fibroblast-like phenotype [102]. This phenotype alters vascular generation and gives rise to pathological wound healing in older adults.

It is important that the ECM of tissues and organs allow immune cells to freely move, patrol, and dwell within tissues for immunological surveillance and protective functions in the absence of peripheral lymphoid organs. Ageing-related alterations in the ECM are significant contributors to immunological ageing. Ageing-related ECM protein glycation alters integrin recognition motifs and directly affects immune cell adhesion and migration. Modest levels of AGE modification in collagen inhibit cell migration by 30%, but high levels lower cell migration by 60% [103]. A rigid ECM directly affects the expression of myosin II and ratios of the nuclear matrix proteins laminin-A, -B, and -C, whereby an increase in laminin-A and -C levels will reduce variability in the nuclear membrane, increase the degree of nuclear compression, and limit the movement of cells [104]. For neutrophils passing through the ageing ECM, the degree of nuclear compression is the highest among different cell types, with the plasma and nuclear membranes being subjected to strong mechanical constraints limiting the mobility of the cell. Forced passage of cells leads to extensive damage to the plasma membrane and nucleus, immune cell dysfunction, and potential cell death. When the clearance capacity of macrophages is exceeded, apoptotic bodies leak, generating a highly inflammatory environment and causing immune senescence [105]. Immunosenescence causes parenchymal cell dysfunction via direct or indirect pathways, and the cells may fail to successfully carry out immunological monitoring and protection. Ageing-related ECM alterations slow angiogenesis and cause a vicious loop in the context of ageing and ECM modification. In addition, active ECM breakdown products have chemotactic effects on monocytes. For example, fragments of collagen, elastin, FN, and laminin produced during ECM degradation contain unique bioactive structural sites, including the tripeptide GHK (collagen Iα2 fragment) and the peptide κ-elastin (elastin fragment). These sites, defined as stromal factors, exhibit chemotactic effects on monocytes in the tissue microenvironment. These stromal factors induce monocyte differentiation into macrophages, which may be further enhanced by dysregulated ECM degradation in response to the secretory phenotype of senescent cells.

## Therapeutic strategies for promoting angiogenesis by targeting the ECM

### Clinical diseases

The morphology and composition of blood vessels in various tissues change with age. In older individuals, significant thickening of the vascular basement membrane, smaller lumens, increased vascular stiffness, decreased compliance, and weakening of contractile function in blood vessels are observed, resulting in a gradual increase in the diameter of blood vessels or the formation of circuitous rings. These alterations result in a reduction in arterial perfusion, damage to the vascular wall, and the development of atherosclerosis and other pathologies. Evidence from animal studies with hindlimb ischaemia suggests that when major arteries are occluded, the ability to protect tissues from ischaemic injury and generate new blood vessels is impaired with ageing, resulting in delayed wound healing and the development of ischaemic disease.

#### Cardiovascular disease

While the pathogenesis of coronary atherosclerosis is extremely complex, the ECM plays an important role, given that the cardiovascular system is rich in ECM. As the vascular ECM changes with ageing, affecting angiogenesis, this may lead to the development of ischaemic cardiovascular disease. In ageing, collagen deposits thicken the vascular basement membrane, ageing-dependent changes in the conformation of elastin lead to increased Ca^2+^ content on elastic fibres [35, 36], and binding of elastin to Ca^2+^ weakens elasticity and increases lipid uptake, which exacerbates atherosclerotic progression [37]. The iso-Asp-Gly-Arg (isoDGR) motif is formed by extensive deamidation of the amino acid sequence Asn-Gly-Arg in ageing-affected FN. The presence of the isoDGR motif causes FN to assume a stretched unfolded state, promoting the formation of protofibrils and deposition of insoluble matrix, resulting in FN accumulation in the basement membrane and thickening of the intima. Ageing-induced FN degradation enhances macrophage activation and monocyte infiltration, which contribute to early atherosclerotic pathological events [104]. The ratio of highly sulphated to hypo-sulphated GAGs increases with age. Highly sulphated GAGs interact with lipoproteins and may be linked to an increase in atherosclerotic risk. The development of atherosclerosis appears to be linked to ageing-related changes in components of the vascular ECM, and the ECM may thus represent a promising biological target for treating ischaemic cardiovascular disease in older adults.

#### Neurological diseases: ischaemic stroke and AD

Cerebrovascular disease is the second largest cause of death worldwide, with a rate 1.5% higher than that of ischaemic heart disease [106]. Ischaemic stroke is caused by a disturbance in blood flow to a portion of the brain, which results in an ischaemic cascade of seizures and eventually irreparable brain damage. Ischaemic stroke accounts for approximately 87% of all stroke cases [107]. Age is the single unchangeable risk factor for cerebral ischaemia. In animal models of ischaemic stroke, older rats consistently show more blood–brain-barrier damage than younger rodents, resulting in vasogenic oedema, poorer neurological function, and higher mortality [108, 109]. The potential mechanisms underlying the increased sensitivity of the ageing brain to neurovascular injury are complicated and include increased oxidative damage, increased levels of pro-inflammatory factors, and decreased neovascularisation and neurogenesis due to dysregulation of MMP activity [110]. Activity of MMP-2 and MMP-9 is significantly increased during stroke [111], and this disrupts the balance of ECM degradation and formation, impairs neovascularisation, relaxes the matrix, promotes cell swelling, disrupts the blood–brain barrier, and promotes brain oedema. High MMP levels in the blood during the acute phase increase the risk of secondary haemorrhage and haemorrhagic stroke [112, 113]. Impaired angiogenesis reduces the number and diameter of collateral branches and impairs cerebral collateral circulation, resulting in significantly reduced blood flow to ischaemic tissues, accelerating tissue infarction and oedema, and affecting prognosis after ischaemic brain injury [112, 114].

Changes in the ECM play a central role in neurodegenerative diseases, including AD and Parkinson’s disease. AD is a chronic neurological disease that represents the most common form of dementia in older adults. Patients with advanced AD have significantly lower capillary density in the frontal and temporal lobes, with corresponding changes in ECM components of the vessel wall, including increased levels of type-IV collagen, perlecan, and collagen; thickened basement membranes; and increased levels of MMP-3 and decreased levels of MMP-9 in cerebrospinal fluid. Dysregulation of MMPs leads to reduced basal turnover, accumulation of ECM components, and potential dysfunction of the blood–brain barrier [115].

Many strategies have been used to re-establish blood supply to the brain to address the deleterious effects of ischaemia, primarily the use of anti-thrombotic drugs to improve cerebral blood circulation. In addition, neuroprotective agents have been used to protect neurons from ischaemia-induced death. However, these agents have drawbacks, including non-specific distribution, short half-life, and low targeting efficiency, negatively impacting the effectiveness and increasing the risk of bleeding [116]. The development of novel therapeutic approaches will be essential for effective treatment.

#### Wound healing

Even in healthy individuals, ageing causes delayed wound healing. When older mice were compared to younger mice, re-epithelialisation, collagen synthesis, and angiogenesis were found to be delayed, and wound healing time was increased. Delayed wound healing is a significant global hazard to the health of older adults, particularly in the context of osteoporotic fractures with reduced wound recovery. As discussed, at every step of wound healing, ageing-related changes occur in the injury zone, including delayed re-epithelialisation, decreased angiogenesis, collagen deposition, and collagen remodelling, as well as reduced capillary density. Interestingly, in animal models and clinical investigations, exercise has been observed to promote cutaneous wound healing by lowering levels of pro-inflammatory cytokines in wound tissue [117]; however, the benefit is relatively limited. Several anti-ageing natural products, including quercetin and resveratrol, are now being used to support wound recovery in older adults. Increasing evidence suggests that these molecules promote wound healing in vivo and in vitro; however, information on safety and efficacy remains limited [118]. The potential to stimulate the healing of bone and skin defects during ageing has far-reaching clinical and economic implications. Understanding how ageing-related ECM changes affect angiogenesis may provide insights into the mechanisms underlying delayed wound healing and provide a basis for therapeutic drug discovery and development targeting the ECM for the treatment of chronic wounds.

### Therapeutic strategies

#### Increasing elastin and collagen content

During ageing, an increase in levels of reactive oxygen species, along with other factors, can lead to a decrease in elastin and collagen synthesis, affecting angiogenesis and leading to the development of disease. Therefore, enhancing elastin and collagen content has emerged as a promising therapeutic approach in a variety of diseases.

Increasing the synthesis of collagen can reduce vascular stiffness and promote angiogenesis. When poly-D,L-lactic acid was injected into the skin of older mice, the synthesis of collagen increased significantly, and ECs showed a greater ability to migrate, proliferate, and form vascular tube structures [119]. The use of poly-D,L-lactic acid represents a promising approach to improving organ perfusion. Measures to increase elastic fibre formation in older adults may also ameliorate ageing-related cardiovascular dysfunction and poor organ perfusion. Researchers have recently begun to assess the efficiency of pharmacological treatments aimed at increasing elastin production and elastic fibre assembly, including the use of minoxidil and dill extract (DE). Minoxidil is a K^+^ channel opener that reduces intracellular Ca^2+^ levels by inducing K^+^ efflux and vascular smooth muscle cell hyperpolarisation. This closes voltage-dependent membrane Ca^2+^ channels, thereby stimulating elastin expression while also reducing levels of elastin AGE products, decreasing elastase activity, and reducing ROS production. While minoxidil is considered a potential anti-arterial ageing agent [120], use is limited by side effects, including oedema and heart enlargement [121, 122], and it will be necessary to develop agents with fewer side effects. DE maintains pre-existing elastic lamellae, activates expression of pro-elastin and LOXL-1, stimulates de novo production of elastic fibres, and lowers the stiffness of the ageing aorta wall while correcting ageing-related cardiac hypertrophy [122], offering treatment advantages over minoxidil. However, the mechanism of action remains unknown, and more research is needed, although DE appears to represent a promising new anti-ageing drug for the cardiovascular system.

#### Inhibiting glycation and cross-linking of matrix proteins

Increases in the levels of AGEs and cross-linking of matrix proteins contribute to mechanical changes in ageing-affected ECM. It is well known that AGEs accumulate during normal ageing. Non-enzymatic glycation and cross-linking of matrix proteins increase ECM and vascular stiffness and reduce neovascularisation [123], compromising the structural integrity and physiological function of multi-organ systems [124]. AGEs interact with cell surface RAGE receptors to promote oxidative stress and inflammatory responses and impair angiogenesis. AGEs thus play a role in disease states, including atherosclerosis, cerebrovascular lesions, and wound healing [125]. Disrupting the cross-linking of AGEs and reducing the formation and interaction of matrix protein glycation products may provide a novel approach to promoting angiogenesis in ischaemic contexts in older adults. Researchers have investigated the effectiveness of several drugs aimed at reducing the formation of matrix protein AGEs, including angiotensin receptor blockers, metformin, and aminoguanidine, to determine their ability to prevent the formation of matrix protein AGEs. However, to date, side effects have limited use of these drugs. Natural products with possible anti-glycation effects have attracted considerable attention. For example, anti-oxidant activity and an ability to scavenge reactive carbonyl compounds in a neem (*Azadirachta indica*) leaf extract were shown to effectively suppress the formation of matrix protein AGEs, and this could encourage collateral angiogenesis in the presence of ischaemia [126]. Because AGE-based cross-linking causes vascular sclerosis and cardiovascular injury, preventing such cross-linking may be an effective strategy for minimizing the onset of these ageing-related diseases. ALT-711, a 4,5-dimethylthiazole derivative, was recently shown to disrupt glucose-derived crosslinks in matrix proteins, including collagen, lowering AGE levels by approximately 30% in aged rats [127, 128]. Animal and clinical trials using AGE-disrupting agents, including TRC4186, C16, and KIOM-79, have shown that these disrupt collagen cross-linking and improve collagen solubility [129–131]. However, the precise consequences of treatment with AGE-disrupting drugs remain a matter of debate, and there is a need for additional strategies to minimize matrix protein glycosylation. Encouragingly, an anti-RAGE monoclonal antibody that binds a peptide sequence in the RAGE extracellular structural domain prevented receptor-ligand binding and significantly improved hindlimb perfusion in ischaemic limbs, with greater reconstruction of collateral circulation in occluded arteries [132]. Recombinant proteins containing ELP fused to the RAGE domain or to stromal cell-derived factor 1 can inhibit AGE-RAGE signal transduction and promote cell proliferation and angiogenesis [133]. Blocking the AGE-RAGE-based response involving matrix proteins is emerging as a promising approach for preventing the development of ischaemic diseases in older adults.

#### Promoting matrix homeostasis

As discussed, MMP-2 and MMP-9, which belong to the collagenase group of enzymes capable of dissolving basement membranes, show dysregulated expression and activity in the ageing vascular system and have been linked to illnesses with impaired angiogenesis. The activity is greatly elevated in ischaemic stroke and cardiovascular disorders, contributing directly to poor outcomes. MMP inhibition has been shown to be effective in the treatment of many diseases, although MMP inhibitors typically have off-target effects involving the inhibition of other metalloenzymes. Non-zinc-binding ligands are potential therapeutics; for example, 7-amino-phenanthridin-6-one is a recently reported drug candidate that inhibits MMP-2 and MMP-9 in a non-competitive manner and does not interfere with the activities of other enzymes. The discovery of new drugs of this kind is expected to enhance an understanding of disease progression and improve prognosis by boosting collateral angiogenesis. This drug candidate also demonstrated neuroprotection against oxidative stress while maintaining MMP inhibitor activity, and activity of this kind is critical for the treatment of neurological diseases, including ischaemic stroke and AD [134].

#### Other approaches

Protein tissue engineering techniques have recently emerged as a strategy for treating ischaemic diseases, including the co-design of a novel ECM material for promoting angiogenesis, using biomaterials as carriers of ECM components or angiogenesis-promoting growth factors. Novel biosynthetic materials, including matrix proteins such as laminin, FN, and collagen, or functional elements of these proteins, have shown efficacy in accelerating ischaemic tissue recovery [135–137]. Another approach involves using biomaterials derived from ECM as stents, for example, via decellularisation of human or porcine aortic adventitia. With the addition of basic fibroblast growth factor, it is possible to formulate biomaterials that simulate the microstructure of natural matrices. Basement membrane proteins, including type-IV collagen, laminin, and FN, which promote cell adhesion and influence signalling cascades to modulate cell differentiation and regeneration, are retained in these decellularized scaffolds, which have been shown to be effective in promoting angiogenesis [138]. Recently, cell-assembled ECM has been tested as a biomaterial suitable for promoting tissue regeneration [139]. As this bio-scaffold degrades, growth factors that stimulate stem cell differentiation are released, resulting in a unique milieu conducive to tissue regeneration. ECM bio-scaffolds can be adapted for specific tissue regeneration applications by selecting the biological tissue of origin, density, and delivery mechanism. Such bio-scaffolds are advantageous due to their ability to mimic specific tissues [140]. Angiogenesis in ECM bio-prostheses remains under-investigated, but the ability of ECM-derived bio-prostheses to promote angiogenesis is critical for regenerative medicine applications and warrants further investigation.

## Conclusions and future perspectives

Ageing is a biological process that affects all living beings. Increasing evidence suggests that in humans, ageing-related alterations in the vascular ECM represent one of the mechanisms of impaired angiogenesis. While angiogenesis declines with age, this is not always detrimental. For example, impaired angiogenesis in tumours can impede tumour growth, and some researchers believe that this change is a protective response to the increased occurrence of malignancies with age. Nonetheless, older adults have a high risk of impaired neovascularisation, with an impact on ageing-related disease states. Following an overview of the mechanisms, we present four prospective treatment approaches targeting ageing-related changes in matrix components and cell–cell contacts. These approaches show the potential for promoting angiogenesis in older adults. Knowledge of safety and efficacy remains limited, however, and the development of medicines and materials that target the ECM to treat ischaemic cardiovascular diseases continues. This study contributes further to the understanding of the interactions of ECM in angiogenesis as affected by ageing and aims to provide inspiration for anti-angiogenic therapeutic strategies for several diseases.

## Data Availability

Not applicable.

## Abbreviations

AD: Alzheimer’s disease
AGE: Advanced glycation end product
DE: Dill extract
EC: Endothelial cell
ECM: Extracellular matrix
ELP: Elastin-like polypeptide
FGF-2: Fibroblast growth factor 2
FN: Fibronectin
GAG: Glycosaminoglycan
HA: Hyaluronic acid
HBP: Hexosamine biosynthesis pathway
HSPG: Heparan sulfate proteoglycans
IsoDGR: Iso-Asp-Gly-Arg
ITGB1: Integrin β1
LAMB: Laminin β-chain
LOX: Lysyl oxidase
MMP: Matrix metalloproteinase
MT: Microtubules
MT1-MMP: Membrane type 1 MMP
PDGF: Platelet-derived growth factor
PG: Proteoglycan
RAGE: Receptor for AGE
SASP: Senescence-associated secretory phenotype
SLRP: Small leucine-rich proteoglycan
TGF-β: Transforming growth factor β
TIMP: Tissue inhibitor of metalloproteinases
TNF-α: Tumour necrosis factor-α
TSP: Thrombospondin
VEGF: Vascular endothelial growth factor
VEGFR2: VEGF receptor 2

## References

[1] Hudlicka O, Brown M, Egginton S (1992). Angiogenesis in skeletal and cardiac muscle. Physiol Rev.

[2] Heidenreich PA, Trogdon JG, Khavjou OA (2011). Forecasting the future of cardiovascular disease in the United States: a policy statement from the American Heart Association. Circulation.

[3] Hynes RO (2009). The extracellular matrix: not just pretty fibrils. Science.

[4] Bhadada SV, Goyal BR, Patel MM (2011). Angiogenic targets for potential disorders. Fundam Clin Pharmacol.

[5] Chang W-T, Lin Y-C, Hong C-S (2022). Effects of STAT3 on aging-dependent neovascularization impairment following limb ischemia: from bedside to bench. Aging (Albany NY).

[6] Tilan JU, Zbinden S, Epstein SE (2008). Aging impairs both collateral development and angiogenesis in mice. Cardiovasc Revasc Med.

[7] Zhao L, Chen R, Qiu J (2022). CircCRIM1 ameliorates endothelial cell angiogenesis in aging through the miR-455-3p/Twist1/VEGFR2 Signaling axis. Oxid Med Cell Longev.

[8] Hughes S, Gardiner T, Hu P (2006). Altered pericyte-endothelial relations in the rat retina during aging: implications for vessel stability. Neurobiol Aging.

[9] Lam YT, Lecce L, Yuen SC (2019). Androgens ameliorate impaired ischemia-induced neovascularization due to aging in male mice. Endocrinology.

[10] Dai X, Hummel SL, Salazar JB (2015). Cardiovascular physiology in the older adults. J Geriatr Cardiol.

[11] Lähteenvuo J, Rosenzweig A (2012). Effects of aging on angiogenesis. Circ Res.

[12] Ungvari Z, Tarantini S, Kiss T (2018). Endothelial dysfunction and angiogenesis impairment in the ageing vasculature. Nat Rev Cardiol.

[13] Senger DR, Davis GE (2011). Angiogenesis. Cold Spring Harb Perspect Biol.

[14] Hernandez-Segura A, Nehme J, Demaria M (2018). Hallmarks of cellular senescence. Trends Cell Biol.

[15] Varela-Eirín M, Demaria M (2022). Cellular senescence. Curr Biol.

[16] Pratsinis H, Armatas A, Dimozi A (2013). Paracrine anti-fibrotic effects of neonatal cells and living cell constructs on young and senescent human dermal fibroblasts. Wound Repair Regen.

[17] Buehler MJ (2008). Nanomechanics of collagen fibrils under varying cross-link densities: atomistic and continuum studies. J Mech Behav Biomed Mater.

[18] Jacob MP (2003). Extracellular matrix remodeling and matrix metalloproteinases in the vascular wall during aging and in pathological conditions. Biomed Pharmacother.

[19] Jacob M-P (2006). Matrice extracellulaire et vieillissement vasculaire. Med Sci (Paris).

[20] Guilbert M, Roig B, Terryn C (2016). Highlighting the impact of aging on type I collagen: label-free investigation using confocal reflectance microscopy and diffuse reflectance spectroscopy in 3D matrix model. Oncotarget.

[21] Verzijl N, DeGroot J, Thorpe SR (2000). Effect of collagen turnover on the accumulation of advanced glycation end products. J Biol Chem.

[22] Panwar P, Lamour G, Mackenzie NCW (2015). Changes in structural-mechanical properties and degradability of collagen during aging-associated modifications. J Biol Chem.

[23] Edgar LT, Underwood CJ, Guilkey JE (2014). Extracellular matrix density regulates the rate of neovessel growth and branching in sprouting angiogenesis. PLoS One.

[24] Bailey AJ, Paul RG, Knott L (1998). Mechanisms of maturation and ageing of collagen. Mech Ageing Dev.

[25] Yamauchi M, Sricholpech M (2012). Lysine post-translational modifications of collagen. Essays Biochem.

[26] Birch HL (2018). Extracellular matrix and ageing. Subcell Biochem.

[27] Monnier VM, Mustata GT, Biemel KL (2005). Cross-linking of the extracellular matrix by the maillard reaction in aging and diabetes: an update on "a puzzle nearing resolution". Ann N Y Acad Sci.

[28] Collier TA, Nash A, Birch HL (2018). Effect on the mechanical properties of type I collagen of intra-molecular lysine-arginine derived advanced glycation end-product cross-linking. J Biomech.

[29] Collier TA, Nash A, Birch HL (2015). Preferential sites for intramolecular glucosepane cross-link formation in type I collagen: a thermodynamic study. Matrix Biol.

[30] Brooke B (2003). New insights into elastin and vascular disease. Trends Cardiovasc Med.

[31] Duca L, Blaise S, Romier B (2016). Matrix ageing and vascular impacts: focus on elastin fragmentation. Cardiovasc Res.

[32] Greenwald SE (2007). Ageing of the conduit arteries. J Pathol.

[33] Konova E, Baydanoff S, Atanasova M (2004). Age-related changes in the glycation of human aortic elastin. Exp Gerontol.

[34] Urry D, Trapane T, Sugano H, et al. Sequential polypeptides of elastin: cyclic conformational correlates of the linear polypentapeptide. J AM CHEM SOC. 1981;103(8):2080–2089. 10.1021/ja00398a035.

[35] Tamburro AM, Bochicchio B, Pepe A (2005). The dissection of human tropoelastin: from the molecular structure to the self-assembly to the elasticity mechanism. Pathol Biol (Paris).

[36] Robert L, Robert AM, Fülöp T (2008). Rapid increase in human life expectancy: will it soon be limited by the aging of elastin?. Biogerontology.

[37] Fonck E, Feigl GG, Fasel J (2009). Effect of aging on elastin functionality in human cerebral arteries. Stroke.

[38] Hawes JZ, Cocciolone AJ, Cui AH (2020). Elastin haploinsufficiency in mice has divergent effects on arterial remodeling with aging depending on sex. Am J Physiol Heart Circ Physiol.

[39] Timpl R, Brown JC (1994). The laminins. Matrix Biol.

[40] Hallmann R, Horn N, Selg M (2005). Expression and function of laminins in the embryonic and mature vasculature. Physiol Rev.

[41] Wagner JUG, Chavakis E, Rogg E-M (2018). Switch in Laminin β2 to Laminin β1 isoforms during aging controls endothelial cell functions-brief report. Arterioscler Thromb Vasc Biol.

[42] Lemańska-Perek A, Pupek M, Polańska B (2013). Alterations in molecular status of plasma fibronectin associated with aging of normal human individuals. Clin Biochem.

[43] Antia M, Baneyx G, Kubow KE (2008). Fibronectin in aging extracellular matrix fibrils is progressively unfolded by cells and elicits an enhanced rigidity response. Faraday Discuss.

[44] Wu X-F, Dzenis YA (2007). Size effect in polymer nanofibers under tension. J Appl Phys.

[45] Iozzo RV, Schaefer L (2015). Proteoglycan form and function: a comprehensive nomenclature of proteoglycans. Matrix Biol.

[46] Labat-Robert J, Robert A-M, Robert L (2012). Aging of the extracellular matrix. Médecine & Longévité.

[47] Singh J, Di Ferrante N, Gyorkey F (1977). Plasma glycosaminoglycans in normal individuals of various age. Atherosclerosis.

[48] Järveläinen H, Sainio A, Wight TN (2015). Pivotal role for decorin in angiogenesis. Matrix Biol.

[49] Bailey AJ (2001). Molecular mechanisms of ageing in connective tissues. Mech Ageing Dev.

[50] Li Y, Liu Y, Xia W (2013). Age-dependent alterations of decorin glycosaminoglycans in human skin. Sci Rep.

[51] Järveläinen H, Puolakkainen P, Pakkanen S (2006). A role for decorin in cutaneous wound healing and angiogenesis. Wound Repair Regen.

[52] Mongiat M, Andreuzzi E, Tarticchio G (2016). Extracellular matrix, a hard player in angiogenesis. Int J Mol Sci.

[53] Labat-Robert J, Robert L (2014). Aging of connective tissues: experimental facts and theoretical considerations. Interdiscip Top Gerontol.

[54] West DC, Kumar S (1989). Hyaluronan and angiogenesis. Ciba Found Symp.

[55] Miyamoto I, Nagase S (1984). Age-related changes in the molecular weight of hyaluronic acid from rat skin. Jikken Dobutsu.

[56] Barriere G, Fici P, Gallerani G (2015). Epithelial mesenchymal transition: a double-edged sword. Clin Transl Med.

[57] Peng DH, Ungewiss C, Tong P (2017). ZEB1 induces LOXL2-mediated collagen stabilization and deposition in the extracellular matrix to drive lung cancer invasion and metastasis. Oncogene.

[58] Deng Y, Chakraborty P, Jolly MK (2021). A theoretical approach to coupling the epithelial-mesenchymal transition (EMT) to extracellular matrix (ECM) stiffness via LOXL2. Cancers (Basel).

[59] Imran SAM, Yazid MD, Idrus RBH (2021). Is there an interconnection between epithelial-mesenchymal transition (EMT) and telomere shortening in aging?. Int J Mol Sci.

[60] Zhang J, Jamaluddin M, Zhang Y (2019). Type II epithelial-mesenchymal transition upregulates protein N-Glycosylation to maintain proteostasis and extracellular matrix production. J Proteome Res.

[61] Zhao Y, Zhang J, Sun H (2021). Crosstalk of the IκB Kinase with spliced X-box binding protein 1 couples inflammation with glucose metabolic reprogramming in epithelial-mesenchymal transition. J Proteome Res.

[62] Scott LE, Weinberg SH, Lemmon CA (2019). Mechanochemical signaling of the extracellular matrix in epithelial-mesenchymal transition. Front Cell Dev Biol.

[63] Fattet L, Jung H-Y, Matsumoto MW (2020). Matrix rigidity controls epithelial-mesenchymal plasticity and tumor metastasis via a mechanoresponsive EPHA2/LYN complex. Dev Cell.

[64] Lubarsky B, Krasnow MA (2003). Tube morphogenesis: making and shaping biological tubes. Cell.

[65] Koh W, Sachidanandam K, Stratman AN (2009). Formation of endothelial lumens requires a coordinated PKCepsilon-, Src-, Pak- and Raf-kinase-dependent signaling cascade downstream of Cdc42 activation. J Cell Sci.

[66] Myers KA, Applegate KT, Danuser G (2011). Distinct ECM mechanosensing pathways regulate microtubule dynamics to control endothelial cell branching morphogenesis. J Cell Biol.

[67] Fischer RS, Gardel M, Ma X (2009). Local cortical tension by myosin II guides 3D endothelial cell branching. Curr Biol.

[68] Carrasco-Mantis A, Alarcón T, Sanz-Herrera JA (2023). An in silico study on the influence of extracellular matrix mechanics on vasculogenesis. Comput Methods Programs Biomed.

[69] Ferrari D, Sengupta A, Heo L (2023). Effects of biomechanical and biochemical stimuli on angio- and vasculogenesis in a complex microvasculature-on-chip. iScience.

[70] Lamalice L, Le Boeuf F, Huot J (2007). Endothelial cell migration during angiogenesis. Circ Res.

[71] Ahluwalia A, Jones MK, Szabo S (2014). Aging impairs transcriptional regulation of vascular endothelial growth factor in human microvascular endothelial cells: implications for angiogenesis and cell survival. J Physiol Pharmacol.

[72] Pourheydar B, Biabanghard A, Azari R (2020). Exercise improves aging-related decreased angiogenesis through modulating VEGF-A, TSP-1 and p-NF-Ƙb protein levels in myocardiocytes. J Cardiovasc Thorac Res.

[73] Agah A, Kyriakides TR, Letrondo N (2004). Thrombospondin 2 levels are increased in aged mice: consequences for cutaneous wound healing and angiogenesis. Matrix Biol.

[74] Senger DR, Perruzzi CA, Streit M (2002). The α1β1 and α2β1 integrins provide critical support for vascular endothelial growth factor signaling, endothelial cell migration, and tumor angiogenesis. Am J Pathol.

[75] Geudens I, Gerhardt H (2011). Coordinating cell behaviour during blood vessel formation. Development.

[76] Wang WY, Jarman EH, Lin D (2021). Dynamic endothelial stalk cell-matrix interactions regulate angiogenic sprout diameter. Front Bioeng Biotechnol.

[77] Chen T, Dong J, Zhou H (2020). Glycation of fibronectin inhibits VEGF-induced angiogenesis by uncoupling VEGF receptor-2-c-Src crosstalk. J Cell Mol Med.

[78] Haun F, Neumann S, Peintner L (2018). Identification of a novel anoikis signalling pathway using the fungal virulence factor gliotoxin. Nat Commun.

[79] Hu Q, Moerman EJ, Goldstein S (1996). Altered expression and regulation of the alpha 5beta1 integrin-fibronectin receptor lead to reduced amounts of functional alpha5beta1 heterodimer on the plasma membrane of senescent human diploid fibroblasts. Exp Cell Res.

[80] Reigle KL, Di Lullo G, Turner KR (2008). Non-enzymatic glycation of type I collagen diminishes collagen-proteoglycan binding and weakens cell adhesion. J Cell Biochem.

[81] Hadley JC, Meek KM, Malik NS (1998). Glycation changes the charge distribution of type I collagen fibrils. Glycoconj J.

[82] Paul RG, Bailey AJ (1996). Glycation of collagen: the basis of its central role in the late complications of ageing and diabetes. Int J Biochem Cell Biol.

[83] Di Lullo GA, Sweeney SM, Korkko J (2002). Mapping the ligand-binding sites and disease-associated mutations on the most abundant protein in the human, type I collagen. J Biol Chem.

[84] Knight CG, Morton LF, Peachey AR (2000). The collagen-binding A-domains of integrins alpha(1)beta(1) and alpha(2)beta(1) recognize the same specific amino acid sequence, GFOGER, in native (triple-helical) collagens. J Biol Chem.

[85] Whelan MC, Senger DR (2003). Collagen I initiates endothelial cell morphogenesis by inducing actin polymerization through suppression of cyclic AMP and protein kinase A. J Biol Chem.

[86] Liu Y, Senger DR (2004). Matrix-specific activation of Src and Rho initiates capillary morphogenesis of endothelial cells. FASEB j.

[87] Hoang MV, Whelan MC, Senger DR (2004). Rho activity critically and selectively regulates endothelial cell organization during angiogenesis. Proc Natl Acad Sci U S A.

[88] Waschke J, Baumgartner W, Adamson RH (2004). Requirement of Rac activity for maintenance of capillary endothelial barrier properties. Am J Physiol Heart Circ Physiol.

[89] Kubota Y, Kleinman HK, Martin GR (1988). Role of laminin and basement membrane in the morphological differentiation of human endothelial cells into capillary-like structures. J Cell Biol.

[90] Vernon RB, Sage EH (1995). Between molecules and morphology. Extracellular matrix and creation of vascular form. Am J Pathol.

[91] Davis GE, Senger DR (2005). Endothelial extracellular matrix: biosynthesis, remodeling, and functions during vascular morphogenesis and neovessel stabilization. Circ Res.

[92] Califano JP, Reinhart-King CA (2010). Substrate stiffness and cell area predict cellular traction stresses in single cells and cells in contact. Cel Mol Bioeng.

[93] Krishnan R, Klumpers DD, Park CY (2011). Substrate stiffening promotes endothelial monolayer disruption through enhanced physical forces. Am J Physiol Cell Physiol.

[94] Andresen Eguiluz RC, Kaylan KB, Underhill GH (2017). Substrate stiffness and VE-cadherin mechano-transduction coordinate to regulate endothelial monolayer integrity. Biomaterials.

[95] Lindblom P, Gerhardt H, Liebner S (2003). Endothelial PDGF-B retention is required for proper investment of pericytes in the microvessel wall. Genes Dev.

[96] Chen J, Sivan U, Tan SL (2021). High-resolution 3D imaging uncovers organ-specific vascular control of tissue aging. Sci Adv.

[97] Davis GE, Norden PR, Bowers SLK (2015). Molecular control of capillary morphogenesis and maturation by recognition and remodeling of the extracellular matrix: functional roles of endothelial cells and pericytes in health and disease. Connect Tissue Res.

[98] Stratman AN, Malotte KM, Mahan RD (2009). Pericyte recruitment during vasculogenic tube assembly stimulates endothelial basement membrane matrix formation. Blood.

[99] Saunders WB, Bohnsack BL, Faske JB (2006). Coregulation of vascular tube stabilization by endothelial cell TIMP-2 and pericyte TIMP-3. J Cell Biol.

[100] Andriotis OG, Elsayad K, Smart DE (2019). Hydration and nanomechanical changes in collagen fibrils bearing advanced glycation end-products. Biomed Opt Express.

[101] Freitas-Rodríguez S, Folgueras AR, López-Otín C (2017). The role of matrix metalloproteinases in aging: tissue remodeling and beyond. Biochim Biophys Acta Mol Cell Res.

[102] Jang M, Oh SW, Lee Y (2022). Targeting extracellular matrix glycation to attenuate fibroblast activation. Acta Biomater.

[103] Haucke E, Navarrete-Santos A, Simm A (2014). Glycation of extracellular matrix proteins impairs migration of immune cells. Wound Repair Regen.

[104] Park JE, JebaMercy G, Pazhanchamy K (2021). Aging-induced isoDGR-modified fibronectin activates monocytic and endothelial cells to promote atherosclerosis. Atherosclerosis.

[105] Moreau J-F, Pradeu T, Grignolio A (2017). The emerging role of ECM crosslinking in T cell mobility as a hallmark of immunosenescence in humans. Ageing Res Rev.

[106] Benjamin EJ, Virani SS, Callaway CW (2018). Heart disease and stroke statistics-2018 update: a report from the American Heart Association. Circulation.

[107] Donkor ES (2018). Stroke in the 21st Century: a snapshot of the burden, epidemiology, and quality of life. Stroke Res Treat.

[108] Tan Z, Lucke-Wold BP, Logsdon AF (2015). Bryostatin extends tPA time window to 6 h following middle cerebral artery occlusion in aged female rats. Eur J Pharmacol.

[109] Yu P, Venkat P, Chopp M (2019). Deficiency of tPA exacerbates white matter damage, neuroinflammation, glymphatic dysfunction and cognitive dysfunction in aging mice. Aging Dis.

[110] Candelario-Jalil E, Paul S (2021). Impact of aging and comorbidities on ischemic stroke outcomes in preclinical animal models: a translational perspective. Exp Neurol.

[111] Clark AW, Krekoski CA, Bou S-S (1997). Increased gelatinase A (MMP-2) and gelatinase B (MMP-9) activities in human brain after focal ischemia. Neurosci Lett.

[112] Ma J, Ma Y, Shuaib A (2020). Impaired collateral flow in pial arterioles of aged rats during ischemic stroke. Transl Stroke Res.

[113] Castellanos M, Leira R, Serena J (2003). Plasma metalloproteinase-9 concentration predicts hemorrhagic transformation in acute ischemic stroke. Stroke.

[114] Faber JE, Zhang H, Lassance-Soares RM (2011). Aging causes collateral rarefaction and increased severity of ischemic injury in multiple tissues. Arterioscler Thromb Vasc Biol.

[115] Lepelletier F-X, Mann DMA, Robinson AC (2017). Early changes in extracellular matrix in Alzheimer's disease. Neuropathol Appl Neurobiol.

[116] Kaviarasi S, Yuba E, Harada A (2019). Emerging paradigms in nanotechnology for imaging and treatment of cerebral ischemia. J Control Release.

[117] Keylock KT, Vieira VJ, Wallig MA (2008). Exercise accelerates cutaneous wound healing and decreases wound inflammation in aged mice. Am J Physiol Regul Integr Comp Physiol.

[118] Thanapaul RJRS, Shvedova M, Shin GH (2021). An insight into aging, senescence, and their impacts on wound healing. Adv Geriatr Med Res.

[119] Oh S, Seo SB, Kim G (2023). Poly-D,L-Lactic acid stimulates angiogenesis and collagen synthesis in aged animal skin. Int J Mol Sci.

[120] Fhayli W, Boyer M, Ghandour Z (2019). Chronic administration of minoxidil protects elastic fibers and stimulates their neosynthesis with improvement of the aorta mechanics in mice. Cell Signal.

[121] Slove S, Lannoy M, Behmoaras J (2013). Potassium channel openers increase aortic elastic fiber formation and reverse the genetically determined elastin deficit in the BN rat. Hypertension.

[122] Coquand-Gandit M, Jacob M-P, Fhayli W (2017). Chronic treatment with minoxidil induces elastic fiber neosynthesis and functional improvement in the aorta of aged mice. Rejuvenation Res.

[123] Francis-Sedlak ME, Moya ML, Huang J-J (2010). Collagen glycation alters neovascularization in vitro and in vivo. Microvasc Res.

[124] Yamagishi S (2012). Potential clinical utility of advanced glycation end product cross-link breakers in age- and diabetes-associated disorders. Rejuvenation Res.

[125] Hansen LM, Gupta D, Joseph G (2017). The receptor for advanced glycation end products impairs collateral formation in both diabetic and non-diabetic mice. Lab Invest.

[126] Perez Gutierrez RM, de Jesus Martinez Ortiz M (2014). Beneficial effect of Azadirachta indica on advanced glycation end-product in streptozotocin-diabetic rat. Pharm Biol.

[127] Guo Y, Lu M, Qian J (2009). Alagebrium chloride protects the heart against oxidative stress in aging rats. J Gerontol A Biol Sci Med Sci.

[128] Wolffenbuttel BH, Boulanger CM, Crijns FR (1998). Breakers of advanced glycation end products restore large artery properties in experimental diabetes. Proc Natl Acad Sci U S A.

[129] Cheng G, Wang L-L, Qu W-S (2005). C16, a novel advanced glycation endproduct breaker, restores cardiovascular dysfunction in experimental diabetic rats. Acta Pharmacol Sin.

[130] Joshi D, Gupta R, Dubey A (2009). TRC4186, a novel AGE-breaker, improves diabetic cardiomyopathy and nephropathy in Ob-ZSF1 model of type 2 diabetes. J Cardiovasc Pharmacol.

[131] Kim YS, Kim J, Kim C-S (2011). KIOM-79, an inhibitor of AGEs-protein cross-linking, prevents progression of nephropathy in zucker diabetic fatty rats. Evid Based Complement Alternat Med.

[132] Johnson LL, Johnson J, Ober R (2021). Novel receptor for advanced glycation end products-blocking antibody to treat diabetic peripheral artery disease. J Am Heart Assoc.

[133] Kang HJ, Kumar S, Dash BC (2023). Multifunctional elastin-like polypeptide fusion protein coacervates inhibit receptor-mediated proinflammatory signals and promote angiogenesis in mouse diabetic wounds. Adv Wound Care (New Rochelle).

[134] Rocchi D, Blázquez-Barbadillo C, Agamennone M (2021). Discovery of 7-aminophenanthridin-6-one as a new scaffold for matrix metalloproteinase inhibitors with multitarget neuroprotective activity. Eur J Med Chem.

[135] Oshikawa M, Okada K, Kaneko N (2017). Affinity-immobilization of VEGF on laminin porous sponge enhances angiogenesis in the ischemic brain. Adv Healthc Mater.

[136] Onak Pulat G, Gökmen O, Çevik ZBY (2021). Role of functionalized self-assembled peptide hydrogels in in vitro vasculogenesis. Soft Matter.

[137] Assal Y, Mie M, Kobatake E (2013). The promotion of angiogenesis by growth factors integrated with ECM proteins through coiled-coil structures. Biomaterials.

[138] Fu W, Xu P, Feng B (2019). A hydrogel derived from acellular blood vessel extracellular matrix to promote angiogenesis. J Biomater Appl.

[139] Potart D, Gluais M, Gaubert A (2023). The cell-assembled extracellular matrix: a focus on the storage stability and terminal sterilization of this human "bio" material. Acta Biomater.

[140] Fercana GR, Yerneni S, Billaud M (2017). Perivascular extracellular matrix hydrogels mimic native matrix microarchitecture and promote angiogenesis via basic fibroblast growth factor. Biomaterials.

[141] Ungvari Z, Tucsek Z, Sosnowska D (2013). Aging-induced dysregulation of dicer1-dependent microRNA expression impairs angiogenic capacity of rat cerebromicrovascular endothelial cells. J Gerontol A Biol Sci Med Sci.

[142] Koike T, Vernon RB, Gooden MD (2003). Inhibited angiogenesis in aging: a role for TIMP-2. J Gerontol A Biol Sci Med Sci.

[143] Cheitlin MD (2009). Effects of age on plasma matrix metalloproteinases (MMPs) and tissue inhibitor of metalloproteinases (TIMPs). Yearbook of Cardiology.

[144] Deng X, Ahluwalia A, Xiong X (2008). S1619 MMP9-mediated upregulation of endostatin and downregulation of VEGF in aging gastric mucosa: novel mechanism for impaired angiogenesis. Gastroenterology.

[145] Meschiari CA, Jung M, Iyer RP (2017). Macrophage overexpression of matrix metalloproteinase-9 in aged mice improves diastolic physiology and cardiac wound healing after myocardial infarction. Am J Physiol Heart Circ Physiol.

[146] Brankovic S, Hawthorne EA, Yu X (2019). MMP12 preferentially attenuates axial stiffening of aging arteries. J Biomech Eng.

[147] Kamei M, Hollyfield JG (1999). TIMP-3 in Bruch's membrane: changes during aging and in age-related macular degeneration. Invest Ophthalmol Vis Sci.

[148] Wagatsuma A (2006). Effect of aging on expression of angiogenesis-related factors in mouse skeletal muscle. Exp Gerontol.

[149] Ahluwalia A, Narula J, Jones MK (2010). Impaired angiogenesis in aging myocardial microvascular endothelial cells is associated with reduced importin alpha and decreased nuclear transport of HIF1 alpha: mechanistic implications. J Physiol Pharmacol.

[150] Aoyagi M, Fukai N, Ogami K (1995). Kinetics of 125I-PDGF binding and down-regulation of PDGF receptor in human arterial smooth muscle cell strains during cellular senescence in vitro. J Cell Physiol.

[151] Drubaix I, Giakoumakis A, Robert L (1998). Preliminary data on the age-dependent decrease in basic fibroblast growth factor and platelet-derived growth factor in the human vein wall and in their influence on cell proliferation. Gerontology.

[152] Reed MJ, Corsa A, Pendergrass W (1998). Neovascularization in aged mice: delayed angiogenesis is coincident with decreased levels of transforming growth factor beta1 and type I collagen. Am J Pathol.

[153] Reed MJ, Edelberg JM (2004). Impaired angiogenesis in the aged. Sci Aging Knowledge Environ.

[154] Shin J-W, Lee E, Han S (2022). Plasma proteomic signature of cellular senescence and markers of biological aging among postmenopausal women. Rejuvenation Res.

